# Annotations

**Published:** 1888-07-14

**Authors:** 


### ANNOTATIONS.

**Profane Demonstrations by Friendly Societies.**

PHILOSOPHERS have never been quite able to settle the question whether the human play which is constantly being enacted on the world's stage is properly to be regarded as a tragedy or as a comedy. They have divided themselves into schools on this point, and the same man will sometimes belong to one school and sometimes to the other. As a contribution towards the strengthening of the "comedy" position, a letter lately received from a well-known secretary to one of the London hospitals is worthy of a little consideration. The writer says:—"The task of those who are engaged in the administration of charitable institutions is certainly not an enviable one. If the management organise a ball, a *matinee*, a dinner, a bazaar, or what not, captious critics are ever ready to denounce this or that method as mischievous, or wrong. But of all the opposers to these various means, the most unreasonable seems to be the objector to the demonstrations and church parades periodically held by friendly societies. . . There are cavillers who view the whole proceeding askance, and vote this annual effort for good an intrusion on their quietude, a discordant noise, and an act of irreverence." Now, no doubt it is desirable that Sunday should be a day of quietness, and we in this country like it to be reverently observed, therein showing ourselves to have some understanding of the seriousness of life. But many of us do not seem to be able to understand that it takes all sorts of people to make a world; and that the world is in no sense the exclusive possession of one sort. The friendly societies march to church in orderly procession, accompanied by a crowd. Is that any more wanting in decency than the marching of a regiment of redcoats, also accompanied by a crowd? The friendly societies have at their head and along the line brass bands, more or less musical but undoubtedly enthusiastic. Is there any more irreverence in the hearty and zealous trumpeting of those well-meaning amateurs than in the more measured sounds of the military band outside the church, or of the pealing organ inside? The friendly societies, mindful of the necessities of hospitals, carry boxes, sometimes attached to long poles, and push them about in every direction, even up to the bed-room windows, with determined energy. But does not the churchwarden in his church and the deacon in his chapel send round the bag or the plate or the box for the very same purpose of obtaining money? and is not his determination to have it, if possible, just as great as that of the less ceremonious but not less resolute friendly society man with his long pole and his deal box at the end of it? It is all a question of a little more or less effusiveness of manner; and those who are seriously perturbed by the good-natured and well-intentioned enthusiasm of the friendly societies should suspect themselves of being either bilious or pharisaical. We incline to think it is mostly the "unco' guid" who make these feeble and somewhat irrational complaints. If those good people are really unhappy about the Sunday demonstrations of the friendly societies there is one way of putting a permanent stop to them. Let all the objectors club together and send cheques to the hospitals of exactly twice the amount raised by the friendly societies' church parades. This simple remedy is an infallible specific for the disease, so long as it is administered with unfailing regularity.

**Nurse Farming.**

A CORRESPONDENT, writing to a provincial contemporary, echoes a complaint which is becoming urgent and very general. He says that trained nurses, however indispensable they may be in certain cases, are not available, because of their costliness. He suggests a "relief society," which shall supply the best nurses to the poor at reduced rates. This opens out a very wide question. Generally speaking, all classes of people have to be content with such things as their purses can pay for. A rich man lives in a more costly house, eats more expensive food, wears dearer clothes, than a poor man. This is recognized as inevitable, and nobody seriously complains. But there seems to be a levelling-up influence in sickness which demands that when the poor man is ill he shall have medical attendance as highly skilled, nursing as thorough, and food and drugs as costly as his richer neighbour. Now that, under existing social arrangements, is impossible. At present it is with the poor man's medical attendance and nursing as it is with his other circumstances, he gets what he can pay for. There is, however, a strong public feeling and sentiment that humanity, especially Christian humanity, cannot be satisfied with this. The best conscience of this country demands that there shall be no respect of persons among the sick. When the poor man or woman is seized upon by a dangerous disease, the best medical skill and the most assiduous and competent nursing must hasten to the rescue. We are entirely at one with this sentiment, and we think it is the beginning of an improved state of feeling between the different classes, which may grow and deepen until practical Christianity has permeated the whole social mass in a measure previously unknown. But when we come to the carrying-out of the sentiment in actual life, the difficulties are almost insuperable. It is not merely artizans and small tradesmen who cannot afford trained nurses. Clergymen, doctors in poor neighbourhoods, the higher ranks of clerks, are all in the same hand-to-mouth condition when sickness comes, and cannot spare the needful outlay for the best attendance. What is to be done? There is little or no competition in the nursing market to reduce the rate of charge. The whole body of trained nurses is more or less farmed out either by hospitals or private associations. Can the hospitals or private associations suggest any way out of the difficulty? If trained nurses were multiplied fourfold, useful and necessary work would probably be found for them. Will the problem be solved by beginning new organizations in every parish on a charitable basis? It may surely be hoped not. The General Hospitals, in large towns and the Cottage Hospitals in smaller ones, should make some effort to deal with the subject. It is to them that the public supplies resources for the use and advantage of the sick and suffering poor, and it is they who will be charged with the responsibility if the lack of skilled medical and nursing help should become conspicuous and a scandal.

**Ill-used Woman.**

SOME lady readers have complained that our charges of extravagance made against women are not founded in fact, and are not deserved. They point out, what we gladly admit, that innumerable instances of self-sacrifice and devotion to duty can be found among the gentler sex, and that especially does the hospital world owe them a deep debt of gratitude. All this is most true and most worthy to be recorded. But when this is freely admitted, it still remains the fact that there are a large number of women of whom little can be said except that they spend their lives for no higher purpose than to seek the most frivolous ways of passing the time and the most costly amusements that lie within their reach. Their methods of dressing and entertaining are a disgrace to creatures who make any pretence to reason. Of woman as woman we speak and think with the utmost respect. We have consistently advocated their claims to all the rights and privileges enjoyed by the sterner sex. We write as we do because we feel that the influence of women over men is great beyond calculation. That influence is mostly used for good and for good only. But in too many instances it first ruins a man's mind and character, and then ruins his business. We desire that all women should be what some women are—the light of the home, the solace and stay of man, the sweet and gentle presence which makes coarseness and vice impossible. Those women who think we speak hardly of their sex should see and admit the faults of their less worthy sisters, and save men the unpleasing necessity of so often pointing them out.

